# Fumarase: A Mitochondrial Metabolic Enzyme and a Cytosolic/Nuclear Component of the DNA Damage Response

**DOI:** 10.1371/journal.pbio.1000328

**Published:** 2010-03-09

**Authors:** Ohad Yogev, Orli Yogev, Esti Singer, Eitan Shaulian, Michal Goldberg, Thomas D. Fox, Ophry Pines

**Affiliations:** 1 Department of Microbiology and Molecular Genetics, IMRIC, Faculty of Medicine, Hebrew University, Jerusalem, Israel; 2 Department of Biochemistry and Molecular Biology, IMRIC, Faculty of Medicine, Hebrew University, Jerusalem, Israel; 3 Department of Genetics, The Institute of Life Sciences, The Hebrew University, Jerusalem, Israel; 4 Department of Molecular Biology and Genetics, Cornell University, Ithaca, New York, United States of America; Harvard Medical School, United States of America

## Abstract

In eukaryotes, fumarase (FH in human) is a well-known tricarboxylic-acid-cycle enzyme in the mitochondrial matrix. However, conserved from yeast to humans is a cytosolic isoenzyme of fumarase whose function in this compartment remains obscure. A few years ago, FH was surprisingly shown to underlie a tumor susceptibility syndrome, Hereditary Leiomyomatosis and Renal Cell Cancer (HLRCC). A biallelic inactivation of FH has been detected in almost all HLRCC tumors, and therefore FH was suggested to function as a tumor suppressor. Recently it was suggested that FH inhibition leads to elevated intracellular fumarate, which in turn acts as a competitive inhibitor of HPH (HIF prolyl hydroxylase), thereby causing stabilization of HIF (Hypoxia-inducible factor) by preventing proteasomal degradation. The transcription factor HIF increases the expression of angiogenesis regulated genes, such as VEGF, which can lead to high microvessel density and tumorigenesis. Yet this mechanism does not fully explain the large cytosolic population of fumarase molecules. We constructed a yeast strain in which fumarase is localized exclusively to mitochondria. This led to the discovery that the yeast cytosolic fumarase plays a key role in the protection of cells from DNA damage, particularly from DNA double-strand breaks. We show that the cytosolic fumarase is a member of the DNA damage response that is recruited from the cytosol to the nucleus upon DNA damage induction. This function of fumarase depends on its enzymatic activity, and its absence in cells can be complemented by high concentrations of fumaric acid. Our findings suggest that fumarase and fumaric acid are critical elements of the DNA damage response, which underlies the tumor suppressor role of fumarase in human cells and which is most probably HIF independent. This study shows an exciting crosstalk between primary metabolism and the DNA damage response, thereby providing a scenario for metabolic control of tumor propagation.

## Abstract 2

Upon DNA damage, a cytosolic form of the mitochondrial enzyme fumarase moves into the nucleus where, by virtue of its enzymatic activity, it participates in the cell's response to DNA damage. This potentially explains its known role as a tumor suppressor.

## Author Summary

Fumarate hydratase (FH; also known as fumarase) is an enzyme found in both the cytoplasm and mitochondria of all eukaryotes. In mitochondria, FH is involved in generating energy for the cell through a metabolic pathway called the Krebs cycle. Its role in the cytoplasm, however, is unclear. FH can function as a tumor suppressor: its absence is linked to the formation of human kidney tumors in a syndrome termed HLRCC. We show here that the cytoplasmic version of FH has an unexpected role in repairing DNA double-strand breaks in the nucleus. This role involves the movement of FH from the cytoplasm into the nucleus and depends on its enzymatic activity. Strikingly, when FH is absent from cells, its function in DNA repair can be substituted by high concentrations of one of the enzyme's products, fumaric acid. Our findings imply that FH deficiency leads to cancer because there is not enough fumaric acid in the nucleus to stimulate repair of DNA double-strand breaks; the persistence of these breaks is believed to provoke cancer. The study thus makes a surprising connection between primary metabolism and the cell's response to DNA damage.

## Introduction

It is well documented that single eukaryotic genes can give rise to proteins that are localized to several subcellular locations [1]. This can be achieved at the level of transcription, splicing, translation, and even by a single translation product. In eukaryotes, the enzyme fumarase (also called fumarate hydratase, FH, in higher eukaryotes) is known to participate in the TCA (tricarboxylic acid) cycle in the mitochondrial matrix. However, a common theme, conserved from yeast to humans, is the existence of a cytosolic isoenzyme of fumarase [2],[3]. Cytosolic yeast fumarase was suggested to participate as a scavenger of fumarate from the urea cycle and catabolism of amino acids [4],[5], yet this has never really explained the evolutionary conserved high levels of the protein in the cytosol. In *S. cerevisiae*, the *FUM1* gene is expressed as a single translation product, which is distributed between the cytosol and the mitochondria via a unique mechanism. Our studies indicate that rapid folding of fumarase impedes its import to mitochondria, thereby providing the driving force for retrograde movement of the processed protein back to the cytosol through the translocation pore (our publications referred to in the review by Karniely and Pines above: [6]–[12]). In human, as in yeast, a single gene encodes FH but the mechanism of its distribution between the cytosol and mitochondria (about 50% in each) is still unknown.

A few years ago, FH was surprisingly shown to underlie a tumor susceptibility syndrome, Hereditary Leiomyomatosis and Renal Cell Cancer (HLRCC). This syndrome is characterized by benign cutaneous and uterine leiomyomas, renal cell carcinomas, and uterine leiomyosarcomas [13],[14]. A bi-allelic inactivation of FH has been detected in almost all HLRCC tumors, and therefore FH was suggested to function as a tumor suppressor [13],[14]. However, the link between the TCA cycle defect and tumorigenesis appeared obscure. Recently, it was shown that FH inhibition leads to elevated intracellular fumarate, which in turn acts as a competitive inhibitor of HPH (HIF prolyl hydroxylase) that stabilizes HIF (Hypoxia-inducible factor) by preventing its proteasomal degradation. The transcription factor HIF increases the expression of angiogenesis regulated genes, such as VEGF [15]–[18]. These data suggested a fumarate-dependent tumorigenesis mediated by the regulation of HPH activity and HIF protein levels. Yet this mechanism does not fully explain the necessity of a large cytosolic population of fumarase molecules.

The main objective of this research was to determine the function of cytosolic fumarase isoenzymes in yeast and human cells and thereby contribute to the understanding of its tumor suppressor activity in human cells. We show that cytosolic fumarase is a DNA damage response protein that is recruited from the cytosol to the nucleus following damage. This function of fumarase depends on its enzymatic activity and its absence in cells can be complemented by high concentrations of fumaric acid. Our results indicate that cytosolic fumarase is involved in recognition and recovery from DNA damage, mainly from DNA double-strand breaks (DSBs). This suggests a new link between fumarase deficiency and tumorigenesis, which is probably HIF independent.

## Results

### An Exclusive Mitochondrial Expression of Fumarase in Yeast

As referred to above, fumarase dual distribution between the cytosol and mitochondria is conserved in all eukaryotes studied, including yeast and humans. In mitochondria of all cell types, fumarase functions as an enzyme of the TCA cycle. In this regard, human fumarase expressed in yeast displays significant fumarase enzymatic activity and is capable of complementing the FUM1-TCA function in a Δ*fum1* chromosomal deletion (unpublished data and this study). Nevertheless, the fact that all eukaryotic cells preserve a cytosolic population of the isoenzyme implies conservation of an additional function for this population.

To identify the cytosolic function of fumarase in yeast, we used the two-step total gene synthesis method [19] in order to express fumarase from the mitochondrial genome such that there is no doubt of cytosolic depletion. The method includes the usage of transformation and homologous recombination in both the nuclear and mitochondrial genomes. Yeast strains were constructed in which the chromosomal copy of *FUM1* is deleted, while the identical protein is expressed from a novel mitochondrially coded version of the *FUM1* gene, termed *FUM1^m^*, inside the organelle. Thus, we created the *FUM1^m^* gene utilizing the yeast mitochondrial genetic code to specify fumarase. This gene was separately inserted into otherwise complete mitochondrial genomes, at an ectopic locus flanked by the *COX2* promoter and 5′-UTR, as well as the *COX2* 3′-UTR, as previously described [20],[21]. This strain also contains a nuclear Δ*fum1* null mutation and is designated Δ*fum1/cox2*::*FUM1^m^*, hereinafter referred to as *FUM1^m^*.

**Figure 1:**
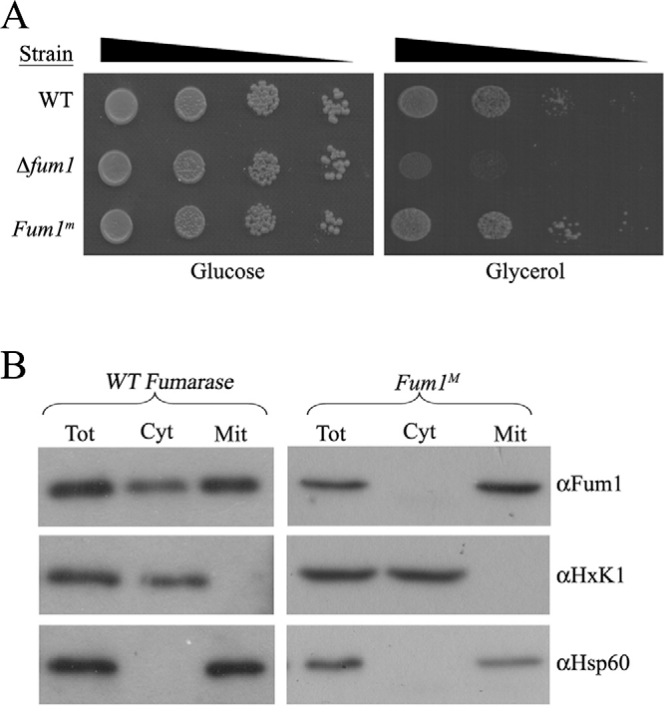
Expression of fumarase from the mitochondrial genome. (A) Mitochondrial encoded fumarase is enzymatically active. WT, *Δfum1*, and *Δfum1/Fum1^m^* (*Fum1^m^*) strains were serially diluted (10^−1^, 10^−2^, 10^−3^, 10^−4^) and grown on fermentable, glucose or non-fermentable, glycerol medium. (B) Mitochondrial encoded fumarase is exclusively localized to mitochondria. WT and *Fum1^m^* strains were subjected to subcellular fractionation as previously described [7]. Equivalent portions of the total (Tot), cytosolic (Cyt), and mitochondrial (Mit) fractions were analyzed by Western blotting using the indicated antibodies. Hsp60 and HxK1 were used as markers for the mitochondria and the cytosol, respectively.

This new strain is capable of growing on galactose or on non-fermentable carbon sources such as glycerol as the sole energy and carbon source, indicating that their mitochondrial TCA cycle is fully functional (Figure 1A and Figure S1). As shown in the subcellular fractionation experiments of Figure 1B, the mitochondrially encoded *FUM1^m^* gene products are localized exclusively to mitochondria as expected (compare cytosolic and mitochondrial fractions).

### Absence of Cytosolic Fumarase Causes Sensitivity to DNA Damage

**Figure 2:**
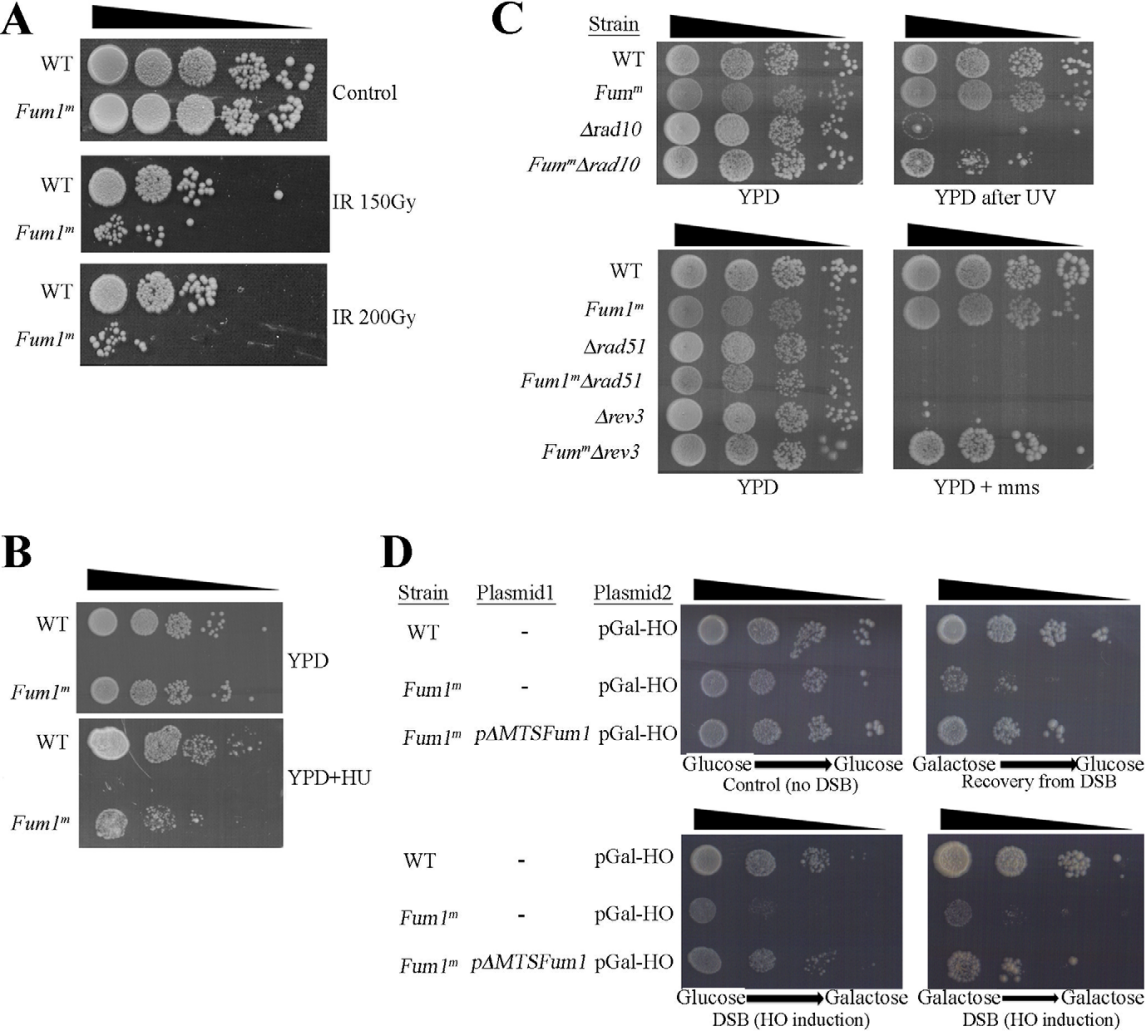
The *Δfum1*/*Fum1^m^* strain is sensitive to DNA damage. (A) WT and *FUM1^M^* were exposed to IR (150 or 200 Gy) and then serially diluted onto YPD plates. (B) Yeast, as above, were serially diluted onto YPD plates containing Hydroxyurea (HU) as indicated. (C) Fumarase cytosolic knockout suppresses the Δ*rev3* and Δ*rad10* phenotypes. Deletion (Δ*rev3*, Δ*rev10*, or Δ*rev51*) and double deletion (Fum1^m^ and Δ*rev3*, Δ*rev10*, or Δ*rev51*) strains were serially diluted and grown on YPD or YPD+MMS plates. Where indicated, plates were irradiated with 10 J/m^2^ UV. (D) WT, *Fum1^m^*, and *Fum1^m^*+*pΔMTS-Fum1* (the latter is a plasmid expressing cytosolic fumarase) were transformed with a plasmid expressing the galactose inducible HO double-stranded DNA endonuclease (pGal-HO). The strains were grown to logarithmic phase in glucose or galactose medium and then serially diluted onto glucose or galactose medium plates as indicated.

Yeast strains lacking cytosolic fumarase *(FUM1^m^*) allow us, for the first time, to study the effect of fumarase absence from the cytosol. Yeast wild type (WT) and *FUM1^m^* strains were tested under various growth conditions including different compositions of growth medium, growth temperature, presence of different growth inhibitors, etc. (unpublished data). Surprisingly, the only significant effect we found was an increased sensitivity (10–100-fold) of the *FUM1^m^* strain to ionizing radiation (IR) (Figure 2A) and to the presence of Hydroxyurea (HU) in the medium (Figure 2B). IR causes DSBs and HU inhibits DNA synthesis. HU is an inhibitor of the enzyme ribonucleotide reductase and treatment with HU blocks the replication fork and ultimately causes chromosome DSBs. These results suggest that in the absence of fumarase from the cytosol, yeast cells are more sensitive to DSBs.

In order to gain some understanding regarding this surprising phenotype, we preformed a genetic analysis of yeast DNA repair pathways. In S. cerevisiae, there are several general groups of repair genes, including nucleotide excision repair (NER), the post-replication repair and the homologous recombination (HR) repair [22]. We created composite mutant strains, which include depletion of cytosolic fumarase (*FUM1^m^*) and the knockout of an essential component of each one of the chosen repair pathways. These strains were tested under different DNA damage conditions (Figure 2C). As expected, a Rad10 knockout strain (Δrad10, NER pathway) is highly sensitive to UV (top panel, row 3) and a Rev3 knockout strain (Δrev3, post-replication repair) is highly sensitive to methyl methanesulfonate (MMS; bottom panel, row 5). Surprisingly, the absence of the cytosolic fumarase in these strains (FUM1^m^-Δrad10 and FUM1^m^- Δrev3) led to less sensitivity to DNA damage treatments (UV and MMS, row 4 top right panel and row 6 bottom right panel, respectively). In contrast, the rad51 knockout strain (Δrad51, HR pathway) is similarly sensitive to MMS in the presence or the absence of cytosolic fumarase (compare Δrad51 to Δfum1/FUM1^m^-Δrad51, rows 3 and 4 of the bottom panels). As a control, when cytosolic fumarase (pΔMTS-FUM1) is expressed in the composite strains, the strains become fully sensitive to their respective treatments (unpublished data), indicating that this phenotype is caused by the absence of cytosolic fumarase. A plausible explanation for these results is that the KO of cytosolic fumarase causes the accumulation of a higher background of DNA damage due to limitation in the function of the HR pathway (to which fumarase presumably belongs). This, in turn, causes up-regulation of other overlapping pathways, which partially suppress mutations in the Rad10 and Rev3 associated pathways. Support for such a notion can be found in the DNA damage response literature in which mutations in the ATM pathway affect the ATR pathway and vice versa [23].

IR and HU can cause DSBs. In order to directly test whether fumarase functions in the response to DSBs, we examined the *FUM1^m^* yeast cell sensitivity to expression of the site-specific double-stranded-DNA endonuclease, HO [24],[25]. This endonuclease put under the GAL promoter (pGAL-HO) allows us to induce a DSB at the HO locus in our strains, not only to test the sensitivity of this strain to DSBs but also to examine the ability of the cells to recover from such damage. WT, *FUM1^m^* and *FUM1^m^* expressing cytosolic fumarase (*p*Δ*MTS-FUM1* from a plasmid) were grown in glucose or galactose media and then plated on glucose or galactose plates. Growth on galactose media, which induces DSBs, allows us to test the sensitivity to DSBs while growth on galactose media followed by growth on glucose (which represses further damage) allows us to test the recovery from the initial DNA damage. As shown in Figure 2D, in the absence of HO (glucose medium, top left panel) both WT and *FUM1^m^* strains grow identically. In contrast, induction of the HO endonuclease (galactose medium, two bottom panels, and glucose after galactose, top right panel) caused a severe inhibition of growth of the *FUM1^m^* strain compared to the WT strain, suggesting that the lack of cytosolic fumarase results in an increased sensitivity of yeast to DSBs and in less efficient recovery from DSBs. Indeed the sensitivity of the *FUM1^m^* strain to DSBs can be almost completely reversed when cytosolic fumarase is co-expressed in this strain (*ΔFUM1^m^*+*pΔMTS-FUM1*) (compare bottom rows of each panel to the middle rows). These results strongly suggest that cytosolic fumarase plays a role in the cellular response to DSBs.

### Fumarase Enzymatic Activity Is Required for Its DNA Damage Protective Function

**Figure 3:**
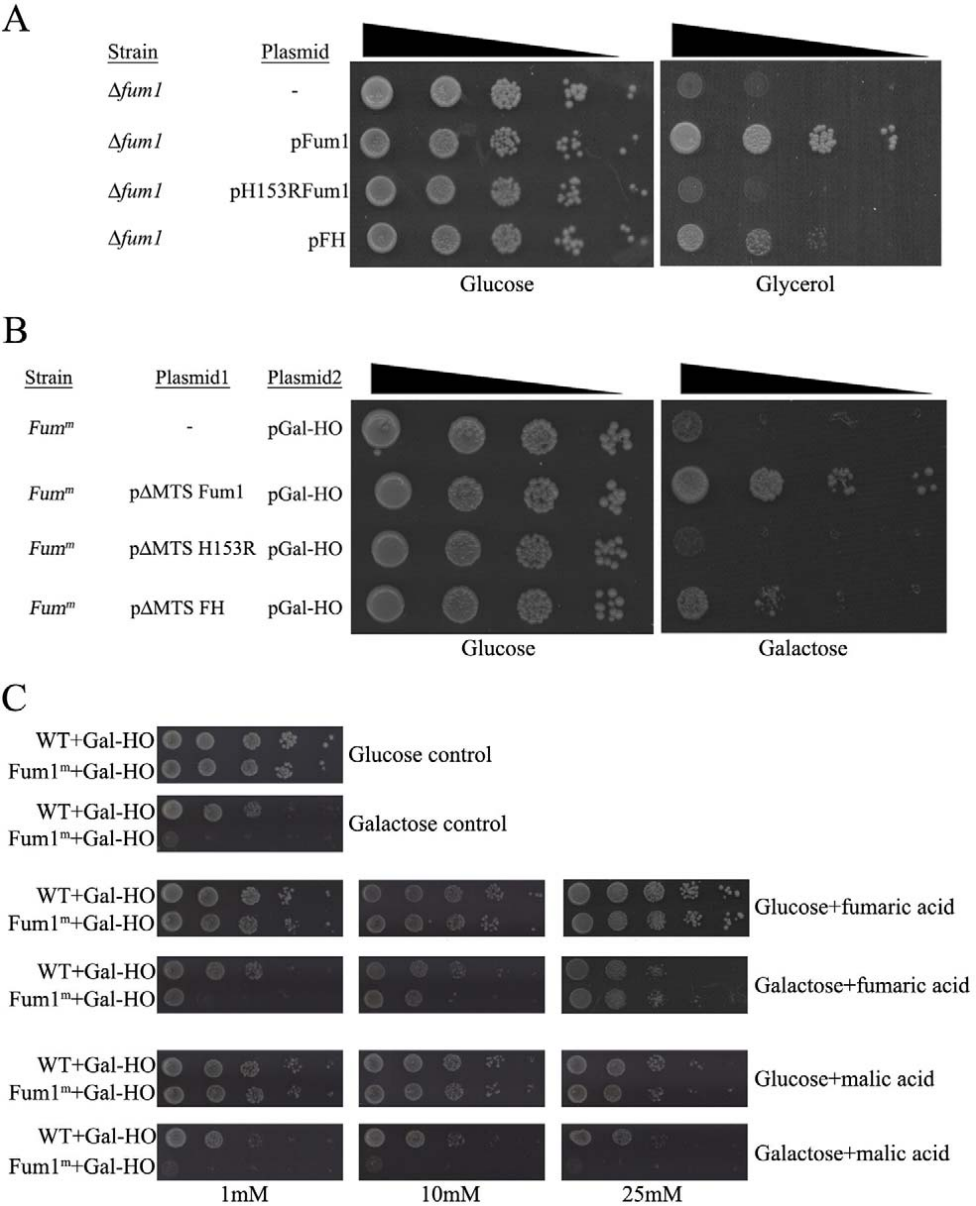
The enzymatic activity of fumarase is crucial for its extra-mitochondrial function. (A) The *Δfum1* strain, harboring the indicated fumarase derivatives, was serially diluted and grown on glucose or glycerol medium plates as indicated. (B) The *Fum1^m^* strain harboring the indicated plasmids and expressing the inducible HO double-stranded DNA endonuclease was serially diluted and grown on glucose or galactose medium plates. (C) High levels of fumarate (but not malate) can suppress the sensitivity of the cytosolic knockout of fumarase to DSBs. WT and *Fum1^M^* strains expressing the inducible HO double-stranded DNA endonuclease were diluted and grown on glucose or galactose medium plates containing 250 mM of phosphate buffer (pH = 8) and the indicated levels of each ester form of the indicated organic acids.

The enzymatic activity of fumarase is obviously required for its function in mitochondria; conversion of fumaric acid to malic acid. Since the DNA-damage-related function of fumarase might be unrelated to this activity, we examined an enzymatically inactive fumarase derivative for its ability to complement the cytosolic function of fumarase. For this, we created a *fum1* point mutation (H153R) that causes a single amino acid change in the enzyme's active site but has no significant effect on its stability [26],[27]. The H153R mutant appears to be catalytically inactive since it does not complement a fumarase knockout strain with respect to its TCA cycle function; yeast strains expressing the mutant protein do not grow on glycerol as the sole energy and carbon source (Figure 3A, compare mutant to the WT, third and second rows, respectively). We next tested whether expression of this mutant derivative in the cytosol could complement the sensitivity to DSBs. The mutant fumarase derivative lacking the MTS was expressed in the *FUM1^m^* strain, expressing the site-specific HO double-stranded DNA endonuclease. Next, yeast were grown on glucose or galactose. As shown in Figure 3B, the *FUM1^m^* strain sensitivity to DSBs can be complemented by catalytically active *p*Δ*MTS-FUM1* but not by the corresponding H153R mutant (compare second and third rows on the galactose plates). Thus, fumarase activity is also required for the extra-mitochondrial function of fumarase.

As pointed out, fumarase catalyzes the reversible conversion of fumaric acid to malic acid. We now wanted to test whether fumaric and/or malic acid, which can be made by the non-mitochondrial fumarase, plays a role in the response to DNA damage. For this, we tested the ability of the yeast *FUM1^m^* strain to grow following induction of DNA damage in the presence of these metabolites. Yeast containing the pGAL-HO (galactose-induced DSB) were grown on glucose media and then plated on glucose or galactose plates containing the indicated concentrations of ester forms of the organic acids (monoethyl-fumarate or diethyl-malate). The use of organic acid esters facilitates their uptake, upon which they are immediately converted to the respective organic acids [28]. As shown in Figure 3C, while the *FUM1^m^* strain is very sensitive to DSB induction and hardly grows on galactose medium (compare the two upper panels), in the presence of 25 mM monoethyl-fumarate the *FUM1^m^* strain grows similarly to the WT (fourth row right panel). Thus, fumaric acid (25 mM) complements the phenotype of fumarase cytosolic absence. In contrast, adding diethyl-malate to the medium had no effect on the protection from DSBs (growth on galactose, bottom panels). This suggests that the role of cytosolic fumarase in the protection against DNA damage is mediated by its catalysis of fumaric acid formation.

A vital question is whether the function in DNA damage response shown for fumarase in yeast is conserved in human cells. We first asked if the lack of cytosolic fumarase in yeast could be complemented by human FH. Human FH is active in yeast and can partially complement the respiratory function of a fumarase knockout strain, allowing the respective yeast to grow on glycerol as the sole energy and carbon source (Figure 3A, fourth row). Moreover, this partial enzymatic activity is sufficient to partially complement the DSBs sensitivity of the *Δfum1*/*FUM1^m^* strain (Figure 3B, fourth row). Taken together, these results strongly support the hypothesis that catalytic activity of fumarase is the key function required in the DNA damage response.

### Fumarase in Yeast and FH in Human Are Induced and Localized to the Nucleus in Response to DNA Damage

**Figure 4:**
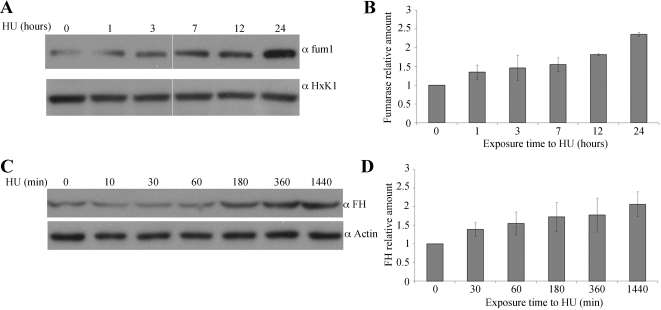
WT yeast fumarase and human FH are overexpressed in response to DNA damage. (A) WT strain was grown to logarithmic phase and HU was added to a final concentration of 300 mM. The levels of fumarase in the cells were determined at the indicated times by Western blot analysis. Anti-HxK1 was used as a loading control. (B) The chart presents the amount of fumarase normalized to the loading control HxK1 and relative to time 0 set at 1. The calculation is based on the optical density of the bands at each time point (*n* = 3; error bars indicate s.d.). (C) HeLa cells were grown in the presence of 1 mM HU for the indicated times. The levels of FH in the cells were determined at the indicated times by Western blot analysis. Anti-Actin was used as a loading control. (D) The chart presents the relative amount of FH, calculated (as above) by measuring the optical density of the FH versus actin bands at each time point (*n* = 3; error bars indicate s.d.).

To examine the effect of DSBs on fumarase expression, yeast were grown to the logarithmic phase, exposed to 300 mM HU for 1, 3, 7, and 24 h and then analyzed by Western blot. As shown in Figure 4A (upper panel), the relative level of fumarase in WT cells increased up to 2.3-fold after 24 h of exposure to HU (compare upper row to the HxK1 loading control below and Figure 4B), indicating that fumarase levels are being regulated by DNA damage.

The main issue at this stage was to test whether the role of fumarase in the DNA damage response is conserved. The fact that FH can complement the cytosolic absence of fumarase in yeast (Figure 3B) supports this notion, and we first analyzed FH levels in human cells after induction of DSBs. HeLa cells were grown in the presence of 1 mM HU for the indicated times (Figure 4C), cell extracts were prepared from these cells, and the levels of FH were analyzed by Western blot. As can be seen in Figure 4C, FH levels increased 2-fold after 24 h (1,440 min) of treatment (Figure 4C, compare the top panel to the actin loading control in the lower panel, and Figure 4D).

**Figure 5:**
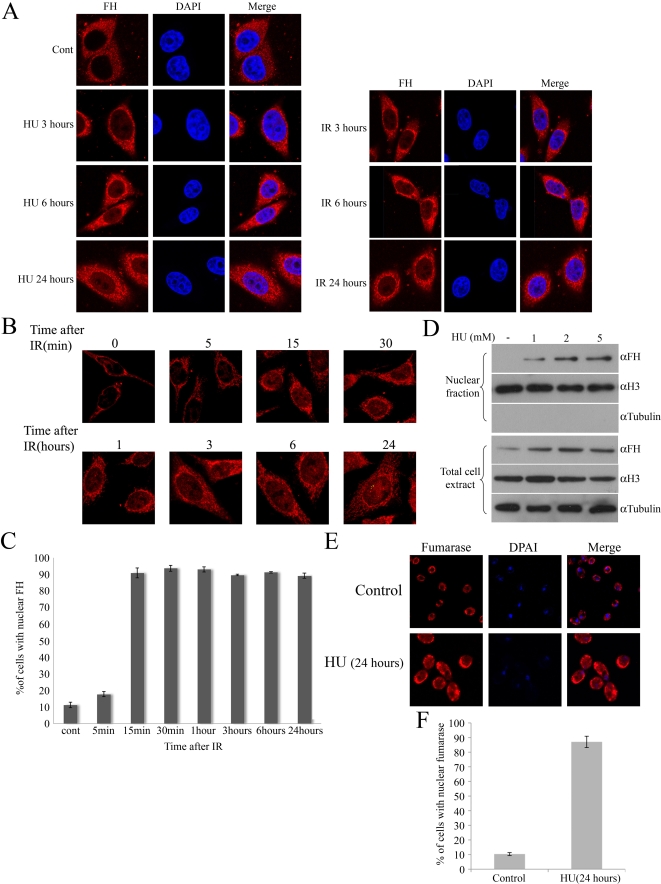
FH is localized in the nucleus after DNA damage. (A) HeLa cells were grown in the presence of HU for 3, 6, and 24 h or irradiated with 5 Gy of IR and left to recover for 3, 6, and 24 h. Cells were fixed, immunostained with anti-FH antibodies and DAPI, and visualized on a confocal microscope. (B) HeLa cells were irradiated with 5 Gy of IR. Cells were fixed and immunostained with anti-FH antibodies and then visualized on a confocal microscope. (C) The charts present the percent of cells of the total cell population containing nuclear FH (*n* = 3; error bars indicate s.d.). (D) HCT cells were grown for 3 h in the presence of the indicated concentration of HU. Nuclear extractions were preformed and analyzed by Western blotting using the indicated antibodies. H3 and Tubulin were used as markers for the nucleus and the cytosol, respectively. (E) WT yeast cells were grown in the presence of HU for 16 h, fixed, and immunostained with anti-fumarase and DAPI. (F) The charts present the percent of cells of the total cell population containing nuclear fumarase staining (*n* = 3; error bars indicate s.d.).

Since the DNA damage response and repair involve activities in the nucleus, we decided to examine FH cellular localization following DNA damage induction. In order to test DSBs induction, we used IR in addition to HU as a DSBs inducer (we do not have the yeast-Gal-HO system in human). In this context the advantage of IR over HU is the option to create DSBs at a specific time and then test recovery of the cells. HeLa cells were examined by immunofluoroscence confocal microscopy after being grown in the presence of HU (1 mM) for 3, 6, and 24 h, or after being exposed to 5 Gy of IR and then left to recover for 3, 6, and 24 h. As shown in Figure 5A (and Figure S2A and S2B), under normal conditions the FH staining pattern reflects its localization mainly to mitochondria and the cytosol. Surprisingly, after both HU or IR treatments, FH is also localized in the nucleus, showing increasing nuclear levels over time.

In order to test the kinetics of FH movement into the nucleus, cells were exposed to IR (5 Gy) and analyzed, as a function of time, by immunofluorescence from early time points following damage induction. As expected, under normal conditions, FH is localized mainly to mitochondria and the cytosol (Figure 5B top left panel). In contrast, 15 min after damage induction, a visible amount of FH staining is localized to the nucleus of treated cells, and this nuclear localization increases over time. This FH nuclear staining was observed in the majority of treated cells (80%–90%) versus 10% of the untreated cells (Figure 5C). In order to get additional biochemical evidence for FH nuclear localization, subcellular fractionation was preformed on HCT cells. Cells were grown in the presence of 1, 2, or 5 mM HU, and nuclear extracts were prepared after 3 h. In contrast to non-treated cells, an increase in nuclear FH is observed as a function of HU dose (Figure 5D).

In order to test whether this re-localization holds true in yeast cells, the WT strain was grown in the presence of HU for 16 h and then examined by immunofluorescence confocal microscopy. As can be seen in Figure 5E and 5F, in the majority of cells fumarase is localized to the nucleus after HU treatment.

### FH Knockdown Increases Sensitivity to DNA Damage of Human Cell Lines

**Figure 6:**
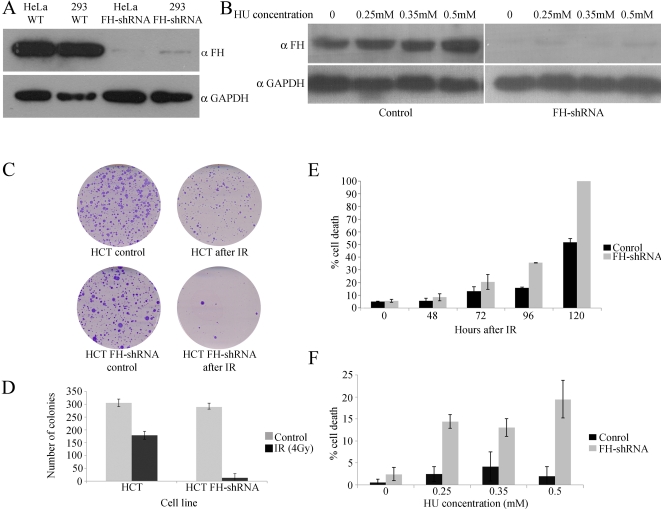
Specific FH shRNA efficiently knockdown the protein expression. (A) HeLa and HEK293 cells were stably transfected with shRNA lentiviral plasmid. FH levels were determined by Western blot analysis. Anti-GAPDH was used as a loading control. (B) HCT116 cells were stably transfected with shRNA lentiviral plasmid and grown in the presence of HU. The levels of FH in the cells at the indicated HU concentrations were determined by Western blot analysis. Anti-GAPDH was used as a loading control. (C–D) FH knockdown cells are sensitive to IR. 1,500 cells were plated per 6 cm plates and incubated for 24 h. Cells were then irradiated with 4 Gy of IR and incubated for 6–8 d. Cells were fixed on the plates using 3.7% formaldehyde, stained with 0.1% crystal violet, and number of colonies was counted (*n* = 3; error bars indicate s.d.). (E) FH knockdown cells are sensitive to IR. HCT116 cells were irradiated with 10 Gy of IR and then incubated for the indicated times. Mortality was determined by Trypan Blue dye exclusion assay (*n* = 3; error bars indicate s.d.). (F) FH knockdown cells are sensitive to HU. HCT116 cells were grown in the presence of indicated concentrations of HU for 48 h. Mortality was determined by Trypan Blue dye exclusion assay (*n* = 3; error bars indicate s.d.).

We next examined whether, as in yeast, absence of FH makes the human cells more sensitive to DNA damage. Since the specific depletion of cytosolic FH, as we achieved in yeast, is not available in human cells, we knocked down total cellular FH expression using a specific shRNA. We generated stable transfected HEK293, HeLa, and HCT116 cell lines expressing the FH shRNA. FH shRNA appears to significantly decrease FH protein levels in all the cells treated (compare the first two lanes to the last two in the upper panels of Figure 6A and Figure 6B). It is worth mentioning that this knockdown does not appear to affect the growth rate of these cells (unpublished data). FH protein levels in these cell lines were very low, and even after HU treatment (which induces FH in control cells) almost no protein was detected (Figure 6B, compare top left and right panels). In order to test cell viability, two approaches were taken. The first was clonogenic assay, in which cells grown at low density are irradiated with 5 Gy and incubated for 6–10 d, after which colonies are stained and counted. As can be seen in Figure 6C and 6D, cells expressing FH-shRNA demonstrated lower amounts of live cells compared to control cells. In the second approach, cell death was tested using Trypan blue staining following exposure to IR or treatment with HU. This assay also demonstrated that cells expressing FH-shRNA are more sensitive to IR (Figure 6E) or HU treatment (Figure 6F). Thus, as in yeast, the expression of FH in human cells is required for survival following DNA damage induction.

### In the Absence of Cytosolic FH, the Cellular Response to DNA Damage Is Impaired

**Figure 7:**
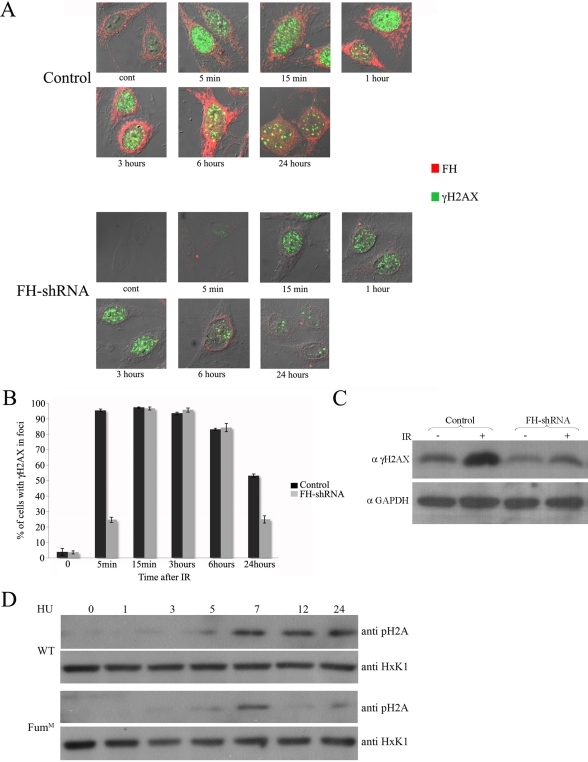
In the absence of FH, timing and levels of histone H2AX phosphorylation are affected. (A) HeLa cells were irradiated with 5 Gy of IR. Cells were fixed and double immunostained with anti-phospho-Ser^139^-H2AX and -FH antibodies. (B) The charts present the percent of cells of the total cell population, presenting foci of phospho-H2AX (*n* = 3; error bars indicate s.d.). (C) Histone H2AX phosphorylation is impaired in knockdown for FH. HCT116 cells were irradiated with 20 Gy of IR and left to recover for 24 h. γH2AX levels were determined by Western blot using anti-phospho-Ser^139^-H2AX antibodies. (D) Histone H2A phosphorylation is impaired in the *Fum1^m^* strain. WT and *Fum1^m^* yeast strains were grown in the presence or absence of HU (300 mM). Phospho H2A levels were determined, after the indicated times, by Western blot using anti-phospho-Ser^129^-H2A antibodies.

One of the earliest responses to DSBs is a rapid and extensive phosphorylation of histone H2AX (or its yeast variant H2A, γH2A(X)). This chromatin modification is highly conserved, but despite its coordinate regulation and its precise role in DSB recognition, its molecular function is not completely understood. γH2A(X) is reported to mediate the recruitment of numerous DSB-recognition and repair factors to the area of the break. Yeast cells unable to generate γH2A are more sensitive to DSBs generation. Histone H2AX null transgenic mice are viable but immunocompromised, IR sensitive, and subjected to increased genomic instability [29]. Using immunofluoroscence we intriguingly found that after IR exposure, phosphorylation of histone H2AX in the HeLa FH-shRNA cells occurs only after 15 min, while in the control cells this phosphorylation occurs already 5 min after IR induction (Figure 7A, compare the upper left panels of the “Control” and “FH-shRNA”). In addition this phosphorylated form of histone H2AX is reduced earlier in the knockdown cells (6 and 24 h after exposure) compared to normal cells (Figure 7A, compare the upper and lower panels, and Figure 7B). This holds true using Western blot analysis, and as shown in Figure 7C, 24 h after exposure to 5 Gy of IR, there are lower levels of the phosphorylated form of the histone (compare the second and fourth bands) in the knockdown FH cells. In order to test whether the same holds true for yeast, WT and *FUM1^M^* strains were grown to the logarithmic phase, exposed to 300 mM HU for 1, 3, 5, 7, and 24 h and then analyzed by Western blot using antibodies against phospho H2A (ser129). As shown in Figure 7D, up until 7 h of exposure, both strains exhibit the same pattern of phosphorylation. Yet after 9 and 24 h, the phosphorylated form of the histone can be found only in WT but not in the *FUM1^M^* strain. This result indicates that in the absence of FH, the recognition of DSBs and/or the DNA damage checkpoint activation are impaired in these cells.

**Figure 8:**
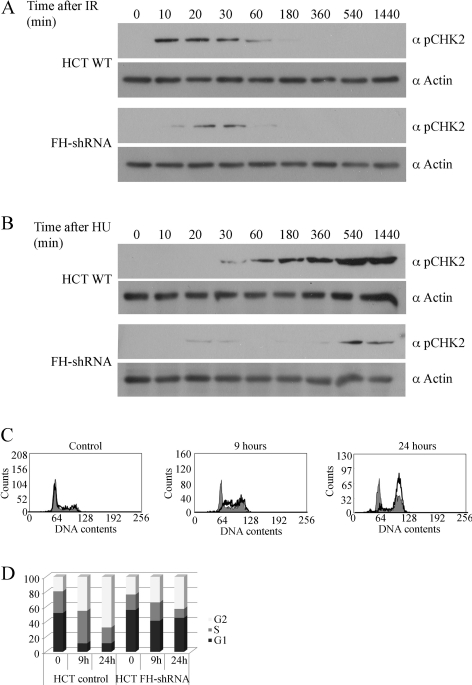
In the absence of FH, the DNA damage checkpoint activation is impaired. HCT cells and FH-shRNA cells were irradiated with 5 Gy (A) or grown in the presence of HU (B) for the indicated times. Phospho-CHK2 levels were determined by Western blot using anti-phospho-Chk2 (Thr^68^) antibodies. (C) HCT WT and FH-shRNA cells were irradiated with 5 Gy of IR and left to recover for the indicated times. Cells were stained with propidium iodide and were analyzed for cell cycle distribution with a flow cytometer (FACS). WT HCT cell cycle histograms are black lined and unfilled, while those of FH-shRNA are filled gray. (D) Relative amounts of cell cycle stages G1 (black), S (gray), and G2 (white) were calculated from the propidium iodide staining shown in (C).

In order to test whether the absence of FH has an effect on checkpoint activation, we tested the phosphorylation of CHK2 (rad53 in yeast), which should be activated upon DSBs induction and regulate cell cycle arrest, DNA repair, and apoptosis [30]. As expected in WT cells, 10 min after IR (Figure 8A, upper panel) and after 30 min of growth in the presence of HU (Figure 8B, upper panel), the phosphorylated form of CHK2 was detected. In contrast, in the FH-shRNA cells much lower levels of phospho-CHK2 were detected and they appeared later after treatment. Next we were interested to ask whether the absence of FH has any affect on cell cycle checkpoint activation after DNA damage. The cell cycles of WT HCT cells and FH-shRNA cells were analyzed by fluorescence-activated cell sorting (FACS). As can be seen in Figure 8C and 8D, WT cells are arrested in G2/S after 9 or 24 h, while the FH knockdown cells do not appear to be arrested (compare middle and left panels, and the graphic presentation). These results support our hypothesis that in the absence of FH, the cellular response to DSB damage is impaired.

## Discussion

Dual targeted proteins represent a problem for simple genetic analysis by gene knockout or by shRNA knockdown, since under such treatments both isoproteins are depleted. The TCA enzyme fumarase (FH) is dual targeted to the cytosol and mitochondria in all eukaryotic cells. While the mitochondrial function of fumarase is well known, the cytosolic function is unclear. Being a scavenger of fumarate made in the urea cycle or from the degradation of certain amino acids could not really explain the evolutionary conserved large amounts of the enzyme in the cytosol. Creation of a yeast strain, in which fumarase is synthesized only within mitochondria, allowed us to screen for unique phenotypes due to the lack of fumarase in the cytosol. Here we report that the absence of fumarase from the cytosol makes the yeast strains more sensitive to DNA damage, mainly to DSBs. This is also true for human FH, as cells knocked down for FH are more sensitive to DSBs. Data supporting this finding include:

(1) Strains of yeast and human cell cultures knocked down for fumarase-FH are sensitive to DNA damage; (2) fumarase in yeast and FH in human cells are induced upon generation of DNA damage; (3) a single amino acid mutation, which abolishes the enzymatic activity of fumarase, renders yeast more sensitive to DSBs; (4) the cellular location of FH is shifted rapidly to the nucleus upon DNA damage induction; (5) cytosolic absence of fumarase in yeast partially suppresses the DNA damage sensitive phenotype of the DNA repair genes, rad10 and rev3; and (6) in the absence of FH, phosphorylation of histone H2AX and of CHK2 and cell cycle checkpoint activation after DSBs induction are impaired.

It was not the initial objective of this study to determine the precise role of fumarase-FH in the DNA damage response. Yet our results may provide some clues to the nature of its extra-mitochondrial activity. Fumarase-FH, according to the phenotypes of its absence, appears to be involved in the cellular response to DNA DSBs. This is supported by the fact that upon induction of DNA damage, FH re-localizes to the nucleus. Notably, it does not accumulate in discrete nuclear foci, at sites of the breaks, as many members of the DNA damage response (Figures 5A and 7A) [31]. Moreover, in the absence of FH, phosphorylation of H2AX and CHK2 and cell cycle checkpoint activation are impaired, suggesting that FH may play a role in the cellular response to DSBs. Important evidence for our current model were the findings that the enzymatic activity of fumarase is essential for its protection from DSBs and that externally added high concentrations of fumaric acid can complement the absence of fumarase in the nucleus/cytosol and protect cells from DSBs. Nevertheless, following IR the whole cell levels of fumaric acid did not change significantly (Figure S3). Taken together these results suggest that FH is not a DNA repair enzyme/protein but rather plays a role in the detection or signaling of DNA damage and in the maintenance of the DSB response machinery. We suggest that FH is doing so by determining the local level of fumaric acid in the nucleus. Unfortunately there is no known way to determine metabolite levels of small molecules such as fumarate or malate in the nucleus. The fact that malic acid cannot and fumaric acid can protect the cells from DSBs brings us to suggest that the conversion of malic to fumaric acid is the relevant function of fumarase in the nucleus. Our finding that there are no significant changes in the whole cell levels of these metabolites following DNA damage suggests that probably the levels of fumaric acid, formed locally by the action of fumarase, in sub-locations of the nucleus are important.

We propose the following model: under normal conditions FH is dual localized in the cytosol and mitochondria where, in the latter, it participates in the TCA cycle. The cytosolic population of FH molecules constitutes a pool, after which the induction of DNA damage (DSBs) is recruited to the nucleus. In the nucleus FH locally produces fumaric acid (from malic acid), which plays a role in the sensing, regulation, and/or stability of the DNA damage response machinery.

The findings indicating that FH, a mitochondrial metabolic enzyme, is a tumor suppressor were fascinating. In this regard, implication of FH in the DNA damage response, in this study, can in essence explain this tumor suppressor activity. In other words, genes involved in the DNA damage response are prime candidates to be tumor suppressors, because in the absence of an intact response (e.g., impaired repair or checkpoint activation) tumorogenic mutations can be established [32]. A recent important report has suggested that in some cases of FH deficiency, there is no accumulation fumarate nor stabilization and accumulation of HIF [33]. This observation can now be explained by a second cellular function of FH in the DNA damage response. We suggest that the major function of FH as a tumor suppressor gene is due to its role in the cellular response to DNA DSBs.

This study shows for the first time an exciting connection between primary metabolism (represented by the enzyme fumarase and its corresponding metabolite, fumaric acid) and the DNA damage response, thereby providing a scenario for metabolic control of tumor propagation. Our findings also support the notion that metabolic enzymes, in addition to being crucial agents of anabolism and catabolism, may play additional, oftentimes central, roles in other cellular activities, as for example aconitase in nucleoid binding and stabilization of mitochondrial DNA [34],[35], aconitase as a regulator of iron metabolism [35], and more recently pyruvate kinase M2 as a phospho-tyrosine binding protein that is critical for a change in metabolism and rapid growth of cancer cells [36].

## Materials and Methods

### Yeast Strains and Growth Conditions

S. Cerevisiae strains used were BY4741 (*Mat α*; *his3Δ1*; *leu2Δ0*; *met15Δ0*; *ura3Δ0*), Δ*rad10* (*Mat α*; *his3Δ1*; *leu2Δ0*; *met15Δ0*; *ura3Δ0;YML095c::kanMX4*), Δ*rad51* (*Mat α*; *his3Δ1*; *leu2Δ0*; *met15Δ0*; *ura3Δ0;YER095w::kanMX4*), Δ*rev3* (*Mat α*; *his3Δ1*; *leu2Δ0*; *met15Δ0*; *ura3Δ0;YPL167c::kanMX4*), *MCC123 rho 0* (*Mat a*; *ade2-101*, *ura3-52*; *kar1-1*; *rho0*), *CAB279-1* (*ade2-101*; *ura3-52*; *kar1-1; fum1::KAN, rho-*), *Δfum1* obtain as previously described [9], Δ*fum1*FUM1^m^ described in this study. The strains Δ*rad10 FUM1^M^*, Δ*rad51 FUM1^M^*, *and* Δ*rev3 FUM1^M^* were created by amplification of the euroscarf knockout cassette including the 5′ and 3′ UTR of each gene by PCR. The resulting PCR products were transformed into Δ*fum1FUM1^m^* and were integrated into the genome, replacing the chromosomal gene.

Strains harboring the appropriate plasmids were grown at 30°C in YPD (1% Yeast extract, 2% peptone, 2% glucose) or synthetic depleted (SD) medium containing 0.67% (wt/vol) yeast nitrogen base +2% glycerol, 2% galactose, or 2% glucose (wt/vol), supplemented with the appropriate amino acids. For agar plates, 2% agar was added. HU was added to YPD to a final concentration of 300 mM, and MMS was added to YPD at final concentration of 0.035%. Monoethyl-fumarate and diethyl-malate were added to glucose or galactose mediums containing 250 mM phosphate buffer pH = 8.

### Cell Lines and Tissue Culture

HeLa and HEK293 cells were grown in DMEM supplemented with 10% fetal bovine serum, and HCT116 cells were grown in McCoy's 5A medium supplemented with 10% fetal bovine serum. Extracts were prepared from cells treated with different doses of IR (using Faxitron Cabinet X-Ray system, model RX-650, Faxitron X-RayCorp.) or from cells treated with the indicated levels of HU.

### Antisera

Polyclonal anti-yeast fumarase and anti-human FH were generated in rabbits injected with the purified proteins. Monoclonal anti-HSP60 (MBL), rabbit polyclonal glucose 6-phosphate dehydrogenase antibodies (Abcam), Polyclonal anti-Hexokinase (Rockland), anti-phospho-Histone H2A (Ser129) -yeast specific (Upstate), Monoclonal anti-phospho-Histone H2A.X (Ser139) (Upstate), Monoclonal anti-actin (MP Biomedicals), monoclonal Lamin A/C (Santa Cruz), anti-phospho Chk2 (Thr68) (Cell Signaling), and anti-H3 (Abcam) were used for immunofluoroscence and immunoblotting as indicated.

### Plasmids

Plasmids encoding WT fumarase (pFT_2_) and the cytosolic fumarase (pIL-ATG1) were described previously [6]. pFT_2_-H153R was created using the QuickChange site-directed mutagenesis kit II (Stratagene). pGal-HO was kindly provided by the laboratory of Giora Simchen (The Hebrew University of Jerusalem). FH knockdown was achieved using MISSION shRNA (SHGLY-NM 000143).

### Yeast Subcellular Fractionation

Yeast cultures were grown on SD medium +2% galactose to 1.5 *A* at 600 nm. Mitochondria were isolated as described previously [7]. Spheroplasts were prepared in the presence of Zymolyase-20T (MP Biomedicals, Irvine, CA). Each of our subcellular fractionation experiments were assayed for cross-contaminations using αHsp60 as a mitochondrial marker and αHexoKinase1 (HxK1) as a cytosolic marker.

### Nuclear Extraction of Human Cells

Cells were harvested with Trypsin, washed twice with PBS, resuspended in buffer A (10 mM Hepes 7.9, 10 mM KCl, 0.1 mM EDTA, 1 mM DTT) and incubated on ice for 15 min. NP-40 was added to final concentration of 0.625% and nuclei were pelleted (7 min at 600 g). The nuclei were washed twice with buffer A, and nuclear proteins were extracted using buffer C (20 mM Hepes 7.9, 0.42 M NaCl, 1 mM EDTA, 1 mM DTT).

### Immunofluorescence Staining of Human Cells

Cells were grown on coverslips and fixed by incubation (30 min) in 4% paraformaldehyde at room temperature (RT). The cells were washed with PBS and incubated with the indicated primary antibody for 1 h at RT in PBS containing 3% bovine serum albumin and 0.3% triton. The slides were then washed with PBS containing 0.1% Tween and then incubated with fluorescently labeled secondary antibody for 40 min at RT. Slides were prepared using dibutylpthalate xylene mounting solution (Fluka Biochemika) and images were visualized using an Olympus or Zeiss confocal microscope. At least 300 randomly selected cells were counted at each point.

### Immunofluorescence Staining of Yeast Cells

Yeast were grown on YPD in the presence or the absence of HU for the indicated times and then fixed by incubation in 3.7% formaldehyde (1 h). Cells were washed and incubated for 5 min in 0.1 M of Tris-SO_4_ and 0.01 M DTT. Cells were then washed using 1.2 M sorbitol and then incubated for 6 min with 1.2 M sorbitol, 0.33 mg/ml Zymolyase-20T, and 10 mM of DTT. Cells were washed twice with sorbitol and placed on cover coverslips (treated with 0.1% polylysine). Slides were immersed in ice-cold methanol for 6 min and then in ice-cold acetone for 30 s. Further immunofluorescence was preformed as above.

### Cell Cycle Assays

Determination of cell cycle was performed at different times following treatment by staining the cells with 5 µg/ml propidium iodide (Sigma) using CyAn ADP (Dako). Both adherent and floating cells were collected for analysis. Cell cycle was analyzed by summit V4.3 software (Dako).

### Metabolite Extraction and Analysis

The cells were collected in equal confluency, by trypsinization, pelleted (1,200×g, 5 min), washed once in PBS, and the pellet (1,200×g, 5 min) was frozen at −80°C until extraction. The cell pellet was suspended in 2.5 ml of boiling 80% (w/w) ethanol and heated for 3 min in a boiling water bath. The samples were then centrifuged and the supernatant recovered. Pellets contained the macromolecules of the cells and were discarded. The supernatants were evaporated in a vacuum drier (SpeedVac). The dry sample was dissolved in 200 µl of deionized water. The solution was filtered through a 0.45 µm filter. The filtrate with the metabolites was then analyzed by anion exchange liquid chromatography with tandem mass spectrometric detection.

## Supporting Information

Figure S1 **Mitochondrial encoded fumarase is enzymatically active and can grow on galactose medium.** WT and δ*fum1/Fum1^m^* (*Fum1^m^*) strains were serially diluted (10^−1^, 10^−2^, 10^−3^, 10^−4^) and grown on glucose (left panel) or galactose (right panel) mediums.
(0.50 MB TIF)

Figure S2 **FH is localized in the nucleus after DNA damage.** HeLa cells were irradiated with 20 Gy of IR and left to recover for 24 h (A) or grown in the presence of 1 mM HU for the indicated times (B). Cells were stained for FH, Hsp60, and DAPI, and pictures were taken through a fluorescent microscope. The graphs present the fraction of cells expressing the nuclear FH from the total cell population (*n* = 3; error bars indicate s.d.).
(7.57 MB TIF)

Figure S3 **Fumaric acid (as well as malic acid) levels in the cell do not change in response to IR.** WT and FH-shFH HeLa cells were exposed to IR and left to recover for 24 h. Metabolite extraction was prepared and analyzed by mass spectrometry as described in the Experimental Procedures section. Presented in the graph is the relative amount of fumaric or malic acid before and after IR treatment.
(0.05 MB JPG)

## Abbreviations

DSBs: double-strand breaks
FACS: fluorescence-activated cell sorting
FH: fumarate hydratase
HIF: Hypoxia-inducible factor
HLRCC: Hereditary Leiomyomatosis and Renal Cell Cancer
HPH: HIF prolyl hydroxylase
HR: homologous recombination
HU: Hydroxyurea
IR: ionizing radiation
MMS: methyl methanesulfonate
NER: nucleotide excision repair
SD: synthetic depleted
RT: room temperature
TCA: tricarboxylic acid
WT: wild type

## References

[1] Karniely S, Pines O (2005). Single translation–dual destination: mechanisms of dual protein targeting in eukaryotes.. EMBO Rep.

[2] Akiba T, Hiraga K, Tuboi S (1984). Intracellular distribution of fumarase in various animals.. J Biochem.

[3] Tuboi S, Suzuki T, Sato M, Yoshida T (1990). Rat liver mitochondrial and cytosolic fumarases with identical amino acid sequences are encoded from a single mRNA with two alternative in-phase AUG initiation sites.. Adv Enzyme Regul.

[4] Ratner S, Anslow W. P, Petrack B (1953). Biosynthesis of urea. VI. Enzymatic cleavage of argininosuccinic acid to arginine and fumaric acid.. J Biol Chem.

[5] Ravdin R. G, Crandall D. I (1951). The enzymatic conversion of homogentisic acid to 4-fumarylacetoacetic acid.. J Biol Chem.

[6] Stein I, Peleg Y, Even-Ram S, Pines O (1994). The single translation product of the FUM1 gene (fumarase) is processed in mitochondria before being distributed between the cytosol and mitochondria in Saccharomyces cerevisiae.. Mol Cell Biol.

[7] Knox C, Sass E, Neupert W, Pines O (1998). Import into mitochondria, folding and retrograde movement of fumarase in yeast.. J Biol Chem.

[8] Sass E, Blachinsky E, Karniely S, Pines O (2001). Mitochondrial and cytosolic isoforms of yeast fumarase are derivatives of a single translation product and have identical amino termini.. J Biol Chem.

[9] Sass E, Karniely S, Pines O (2003). Folding of fumarase during mitochondrial import determines its dual targeting in yeast.. J Biol Chem.

[10] Regev-Rudzki N, Karniely S, Ben-Haim N. N, Pines O (2005). Yeast aconitase in two locations and two metabolic pathways: seeing small amounts is believing.. Mol Biol Cell.

[11] Karniely S, Rayzner A, Sass E, Pines O (2006). Alpha-complementation as a probe for dual localization of mitochondrial proteins.. Exp Cell Res.

[12] Karniely S, Regev-Rudzki N, Pines O (2006). The presequence of fumarase is exposed to the cytosol during import into mitochondria.. J Mol Biol.

[13] Launonen V, Vierimaa O, Kiuru M, Isola J, Roth S (2001). Inherited susceptibility to uterine leiomyomas and renal cell cancer.. Proc Natl Acad Sci U S A.

[14] Tomlinson I. P, Alam N. A, Rowan A. J, Barclay E, Jaeger E. E (2002). Germline mutations in FH predispose to dominantly inherited uterine fibroids, skin leiomyomata and papillary renal cell cancer.. Nat Genet.

[15] Gottlieb E, Tomlinson I. P (2005). Mitochondrial tumour suppressors: a genetic and biochemical update.. Nat Rev Cancer.

[16] Isaacs J. S, Jung Y. J, Mole D. R, Lee S, Torres-Cabala C (2005). HIF overexpression correlates with biallelic loss of fumarate hydratase in renal cancer: novel role of fumarate in regulation of HIF stability.. Cancer Cell.

[17] Pollard P. J, Briere J. J, Alam N. A, Barwell J, Barclay E (2005). Accumulation of Krebs cycle intermediates and over-expression of HIF1alpha in tumours which result from germline FH and SDH mutations.. Hum Mol Genet.

[18] Sudarshan S, Linehan W. M, Neckers L (2007). HIF and fumarate hydratase in renal cancer.. Br J Cancer.

[19] Young L, Dong Q (2004). Two-step total gene synthesis method.. Nucleic Acids Res.

[20] Mireau H, Arnal N, Fox T. D (2003). Expression of Barstar as a selectable marker in yeast mitochondria.. Mol Genet Genomics.

[21] Thorsness P. E, Fox T. D (1993). Nuclear mutations in Saccharomyces cerevisiae that affect the escape of DNA from mitochondria to the nucleus.. Genetics.

[22] Sancar A, Lindsey-Boltz L. A, Unsal-Kacmaz K, Linn S (2004). Molecular mechanisms of mammalian DNA repair and the DNA damage checkpoints.. Annu Rev Biochem.

[23] Hurley P. J, Bunz F (2007). ATM and ATR: components of an integrated circuit.. Cell Cycle.

[24] Dotiwala F, Haase J, Arbel-Eden A, Bloom K, Haber J. E (2007). The yeast DNA damage checkpoint proteins control a cytoplasmic response to DNA damage.. Proc Natl Acad Sci U S A.

[25] Rudin N, Haber J. E (1988). Efficient repair of HO-induced chromosomal breaks in Saccharomyces cerevisiae by recombination between flanking homologous sequences.. Mol Cell Biol.

[26] Alam N. A, Olpin S, Rowan A, Kelsell D, Leigh I. M (2005). Missense mutations in fumarate hydratase in multiple cutaneous and uterine leiomyomatosis and renal cell cancer.. J Mol Diagn.

[27] Kokko A, Ylisaukko-Oja S. S, Kiuru M, Takatalo M. S, Salmikangas P (2006). Modeling tumor predisposing FH mutations in yeast: effects on fumarase activity, growth phenotype and gene expression profile.. Int J Cancer.

[28] MacKenzie E. D, Selak M. A, Tennant D. A, Payne L. J, Crosby S (2007). Cell-permeating alpha-ketoglutarate derivatives alleviate pseudohypoxia in succinate dehydrogenase-deficient cells.. Mol Cell Biol.

[29] Fillingham J, Keogh M. C, Krogan N. J (2006). GammaH2AX and its role in DNA double-strand break repair.. Biochem Cell Biol.

[30] Buscemi G, Carlessi L, Zannini L, Lisanti S, Fontanella E (2006). DNA damage-induced cell cycle regulation and function of novel Chk2 phosphoresidues.. Mol Cell Biol.

[31] Lavin M. F (2008). Ataxia-telangiectasia: from a rare disorder to a paradigm for cell signalling and cancer.. Nat Rev Mol Cell Biol.

[32] Halazonetis T. D, Gorgoulis V. G, Bartek J (2008). An oncogene-induced DNA damage model for cancer development.. Science.

[33] Raimundo N, Ahtinen J, Fumic K, Baric I, Remes A. M (2008). Differential metabolic consequences of fumarate hydratase and respiratory chain defects.. Biochim Biophys Acta.

[34] Chen X. J, Wang X, Kaufman B. A, Butow R. A (2005). Aconitase couples metabolic regulation to mitochondrial DNA maintenance.. Science.

[35] Rouault T. A, Stout C. D, Kaptain S, Harford J. B, Klausner R. D (1991). Structural relationship between an iron-regulated RNA-binding protein (IRE-BP) and aconitase: functional implications.. Cell.

[36] Christofk H. R, Vander Heiden M. G, Wu N, Asara J. M, Cantley L. C (2008). Pyruvate kinase M2 is a phosphotyrosine-binding protein.. Nature.

