# Supplementary figures and images for: Fumarase: A Mitochondrial Metabolic Enzyme and a Cytosolic/Nuclear Component of the DNA Damage Response
Source: PLoS Biol. 2010 Mar 9;8(3):e1000328. doi: 10.1371/journal.pbio.1000328 (PMC2834712; doi:10.1371/journal.pbio.1000328)

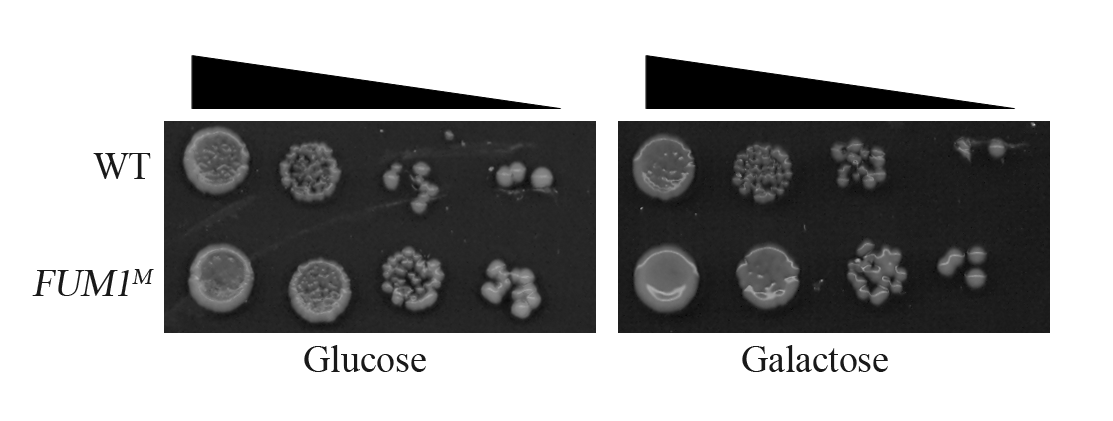

Supplement: Figure S1 — Mitochondrial encoded fumarase is enzymatically active and can grow on galactose medium. WT and δfum1/Fum1m (Fum1m) strains were serially diluted (10−1, 10−2, 10−3, 10−4) and grown on glucose (left panel) or galactose (right panel) mediums. (0.50 MB TIF) [file pbio.1000328.s001.tif]

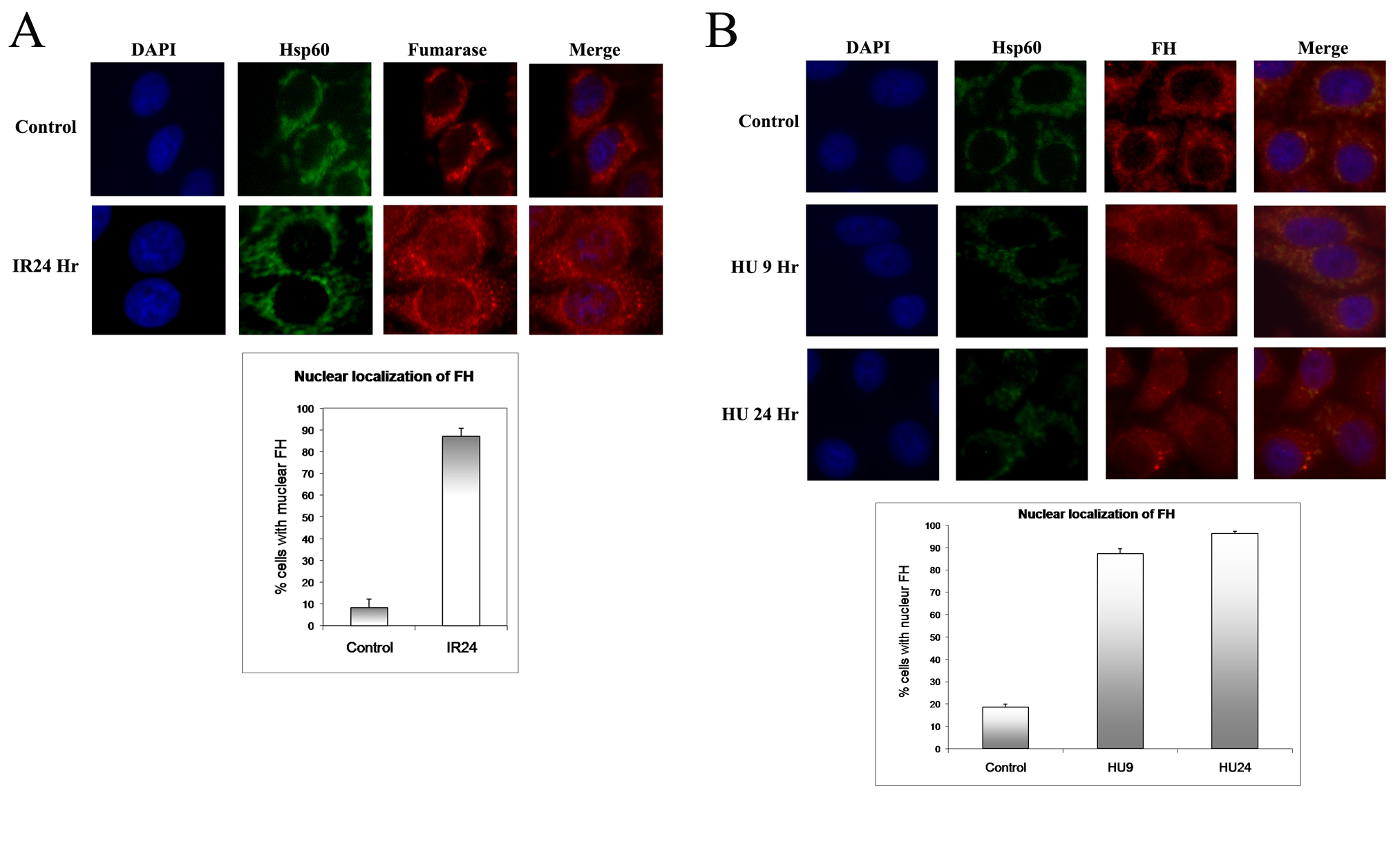

Supplement: Figure S2 — FH is localized in the nucleus after DNA damage. HeLa cells were irradiated with 20 Gy of IR and left to recover for 24 h (A) or grown in the presence of 1 mM HU for the indicated times (B). Cells were stained for FH, Hsp60, and DAPI, and pictures were taken through a fluorescent microscope. The graphs present the fraction of cells expressing the nuclear FH from the total cell population (n = 3; error bars indicate s.d.). (7.57 MB TIF) [file pbio.1000328.s002.tif]

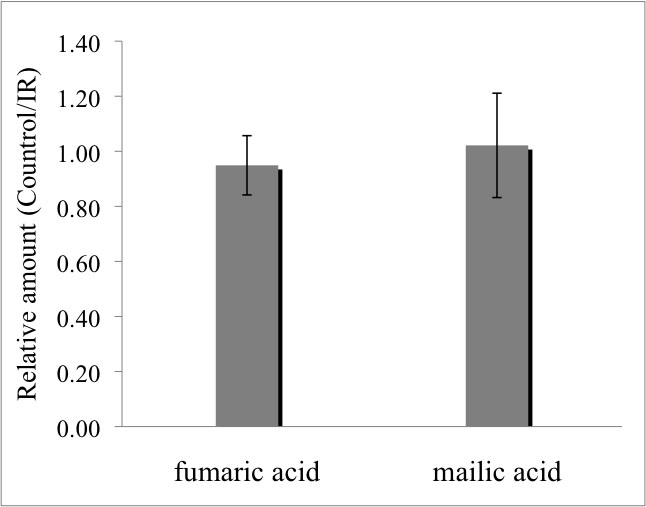

Supplement: Figure S3 — Fumaric acid (as well as malic acid) levels in the cell do not change in response to IR. WT and FH-shFH HeLa cells were exposed to IR and left to recover for 24 h. Metabolite extraction was prepared and analyzed by mass spectrometry as described in the Experimental Procedures section. Presented in the graph is the relative amount of fumaric or malic acid before and after IR treatment. (0.05 MB JPG) [file pbio.1000328.s003.jpg]
